# Metabolic fitness of NAC1-deficient Tregs in the tumor microenvironment fuels tumor growth

**DOI:** 10.1172/jci.insight.186000

**Published:** 2025-01-07

**Authors:** Anil Kumar, Jugal Kishore Das, Hao-Yun Peng, Liqing Wang, Darby Jane Ballard, Yijie Ren, Xiaofang Xiong, Xingcong Ren, Jin-Ming Yang, Paul de Figueiredo, Jianxun Song

**Affiliations:** 1 Department of Microbial Pathogenesis and Immunology, Texas A&M University Health Science Center, Bryan, Texas, USA.; 2 Department of Biochemistry and Biophysics, Texas A&M University, College Station, Texas, USA.; 3 Department of Toxicology and Cancer Biology, Department of Pharmacology and Nutritional Science, and Markey Cancer Center, University of Kentucky College of Medicine, Lexington, Kentucky, USA.; 4 Department of Molecular Microbiology and Immunology, University of Missouri School of Medicine, Columbia, Missouri, USA.

**Keywords:** Immunology, Cancer

## Abstract

The nucleus accumbens-associated protein 1 (NAC1) has recently emerged as a pivotal factor in oncogenesis by promoting glycolysis. Deletion of NAC1 in regulatory T cells (Tregs) has been shown to enhance FoxP3 stability, a suppressor of glycolysis. This study delves into the intriguing dual role of NAC1, uncovering that Treg-specific deletion of NAC1 fosters metabolic fitness in Tregs, thereby promoting tumorigenesis. Our results unveil that NAC1-deficient Tregs exhibited prolonged survival and heightened function, particularly in acidic environments. Mechanistically, we find that NAC1-deficient Tregs adapted to adverse conditions by upregulating FoxP3 expression, engaging in CD36-mediated lipid metabolism, and enhancing peroxisome proliferator–activated receptor gamma coactivator 1-alpha–regulated mitochondrial function. In mouse tumor xenograft models, NAC1-deficient mice demonstrated increased susceptibility to tumor growth. Notably, Tregs lacking NAC1 not only displayed elevated lipid metabolism and mitochondrial fitness but also exhibited enhanced tumoral infiltration. Adoptive Treg transfer experiments further underscored the supportive role of NAC1-deficient Tregs in tumor growth. These findings suggest that modulating NAC1 expression in FoxP3^+^ Tregs could serve as a promising approach to augment antitumor immunity. Understanding the intricate interplay between NAC1 and Tregs opens avenues for potential therapeutic strategies targeting the tumor microenvironment.

## Abstract 2

Deleting NAC1 in regulatory T cells enhances their survival and function, promoting tumor growth by increasing lipid metabolism and mitochondrial fitness, offering potential therapeutic insights.

## Graphical abstract

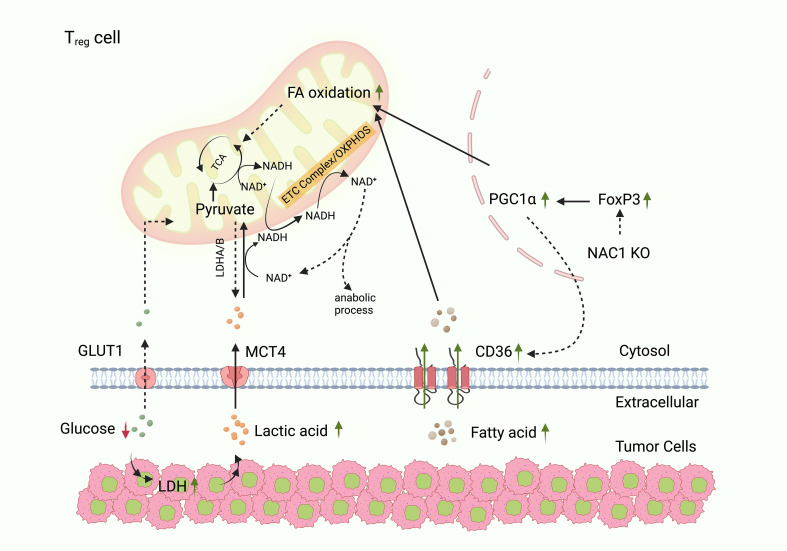

## Introduction

Nucleus accumbens-associated protein 1 (NAC1), a transcription cofactor associated with cancer, is a member of the bric-a-brac tramtrack broad complex/poxvirus and Zn finger family of nuclear proteins. NAC1 overexpression is a feature of several cancer types, including ovarian, cervical, and uterine cancers (1–3), and is believed to promote tumor initiation and progression (4, 5). Furthermore, high expression of NAC1 is closely associated with recurrent ovarian serous carcinoma and contributes to cancer drug resistance (6).

NAC1 is a critical regulator of glycolysis in ovarian cancer development through stabilization of hypoxia-inducible factor-1α, direct regulation of c-Myc in tumor cells, and subsequent regulation of cellular metabolism (7). NAC1 enhances the expression of lactate dehydrogenase A (LDHA) in tumor cells, leading to higher accumulation of lactic acid (LA), which results in altered cellular metabolism of cytotoxic T cells (CTLs) in the tumor microenvironment (TME) (8). It has been shown that T cells can switch to lactate uptake in a glucose-starved TME. However, the reversal of the LDH reaction to generate pyruvate drains the cellular NAD^+^ pools, effectively inhibiting GAPDH activity and glycolytic flux. The nutrient-deficient tumor environment, along with LDHA-mediated LA production, creates a hostile condition for tumor-infiltrating lymphocytes, leading to immune evasion (8–10). FoxP3^+^ regulatory T cells (Tregs), a distinct and dynamic subset of CD4^+^ T cells, are essential for maintenance of immune cell homeostasis (11). Elevated levels of FoxP3^+^ Tregs within the TME showed a positive correlation with poor prognosis in patients with various cancers (12). Foxp3 reprograms T cell metabolism by suppressing c-myc and glycolysis, enhancing oxidative phosphorylation (OXPHOS), and increasing NAD oxidation (13). FoxP3 drives upregulation of components of all the electron transport complexes, increasing their activity and ATP generation by OXPHOS. These adaptations provide a survival and metabolic advantage to Tregs over effector T cells, by generating energy through fatty acid metabolism (OXPHOS) in glucose-deficient and lactate-rich TME (14–17). We recently reported that NAC1 acts as a negative regulator of FoxP3 in Tregs and that NAC1-deficient (NAC1-KO) mice are resistant to autoimmunity and exhibit strong immunosuppressive activity as compared with WT mice (10). We show that NAC1-KO mice generate a higher frequency of CD4^+^ Tregs that exhibit a higher metabolic profile and immunosuppressive activity, exhibit increased acetylation and expression of FoxP3, and exhibit slower turnover of this transcriptional factor (10). Because FoxP3^+^ Tregs are critically involved in tumor development and progression via suppressing antitumor immunity and the elevated number of FoxP3^+^ Tregs in the TME is positively correlated with poor prognosis in patients with cancer (12), we intended to know whether and how NAC1-mediated control of Tregs and their function affect tumorigenesis. Our study found that Tregs with NAC1 deficiency are metabolically more robust and functionally stronger than WT Tregs in the TME, with upregulation of the CD36/peroxisome proliferator–activated receptor gamma coactivator 1-alpha (PGC-1α) pathway, and promote tumor growth by increasing the infiltration of FoxP3^+^ Tregs into tumors. This FoxP3 and CD36 upregulation plays a pivotal role in supporting enhanced lipid metabolism, mitochondrial fitness, and biogenesis within the TME, all through a mechanism dependent on PGC-1α. In an adoptive Treg transfer experiment, we verified that Treg-specific NAC1 deficiency is sufficient to support tumor initiation and growth. These results underscore the potential of targeting metabolic adaptation in intratumoral Tregs as a promising strategy for metabolic reprogramming of the TME.

## Results

### Syngeneic NAC1-KO mice are prone to tumor growth.

**Figure 1:**
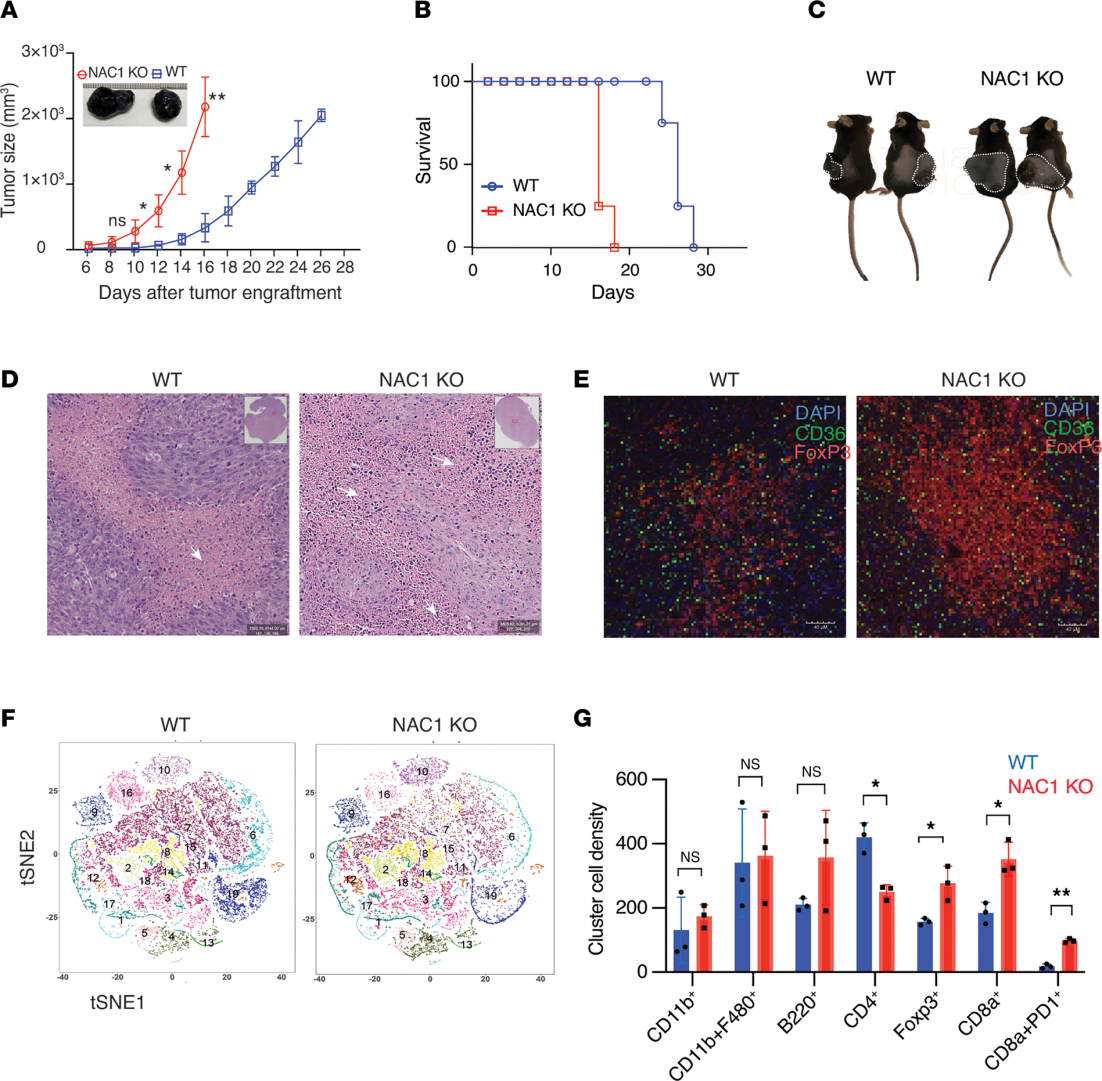
NAC1 deficiency supports tumor growth in mice. B16-F10 melanoma cells (3 × 10^6^ cells/mouse) were s.c. inoculated into WT and NAC1-KO mice, and the tumors were harvested for histology examination. (**A**) Tumor growth curves (*n* = 5). (**B**) Representative survival curve was plotted. (**C**–**E**) Tumors were harvested and examined for the infiltration of lymphocytes by H&E staining (**D**) and FoxP3-expressing Tregs by IHC staining (**E**). Scale bars, 40 µm (**D**); 40 μm (**E**). (**F**) T-distributed stochastic neighbor embedding (t-SNE) plot showing quantification of infiltrating immune cell population in WT versus NAC1-KO generated by IMC analysis performed with the tumor tissue section. (**G**) Quantification of infiltrating immune cell population analyzed by IMC analysis. PD-1, programmed cell death 1. *: *P* ≤ 0.05, **: *P* ≤ 0.01, 2-way ANOVA.

In our previous study, we reported the inhibitory role of NAC1 in FoxP3 expression within Tregs. Given FoxP3’s pivotal role in Treg function and its regulatory nexus with NAC1, we set out to investigate whether NAC1 has any impact on tumor growth. To explore the role of NAC1 in tumor development, we subcutaneously inoculated 3×10^6^ B16-F10 or MC38 tumor cells into the right flank of both WT and NAC1-KO mice, then monitored the progression of the tumors. Remarkably, we observed that B16-F10 cells grew significantly faster and formed larger tumors in NAC1-KO mice than in the age-matched WT mice (Figure 1A), leading to shorter survival of the tumor-bearing mice (Figure 1B). We further examined tumor tissue sections (Figure 1C) for lymphocyte infiltration using hematoxylin and eosin (H&E) staining (Figure 1D), immunofluorescence staining (Figure 1E), and imaging mass cytometry (IMC) analysis (Figure 1, F and G). H&E-stained tissue sections demonstrated an increased lymphocyte infiltration in the MC38 tumors grown in NAC1-KO mice, as compared with those grown in WT animals (Figure 1D). t-SNE plots of the IMC data verified that the total tumoral infiltration of FoxP3^+^ Tregs was significantly higher in NAC1-KO mice than that in WT mice, but the infiltration of total CD4^+^ T cells was significantly higher in WT mice than in NAC1-KO mice (Figure 1G). Also, the infiltration of CD8^+^ and CD8^+^PD-1^+^ T cells was significantly higher in tumors grown in NAC1-KO mice than in tumors grown in WT animals (Figure 1G), suggesting that exhaustion and reduced survival of CD8^+^ T cells may contribute to the enhanced functional fitness of the tumor-infiltrating Tregs in NAC1-KO mice.

### NAC1 negatively affects the survival and polarization of Tregs in acidic environments.

**Figure 2:**
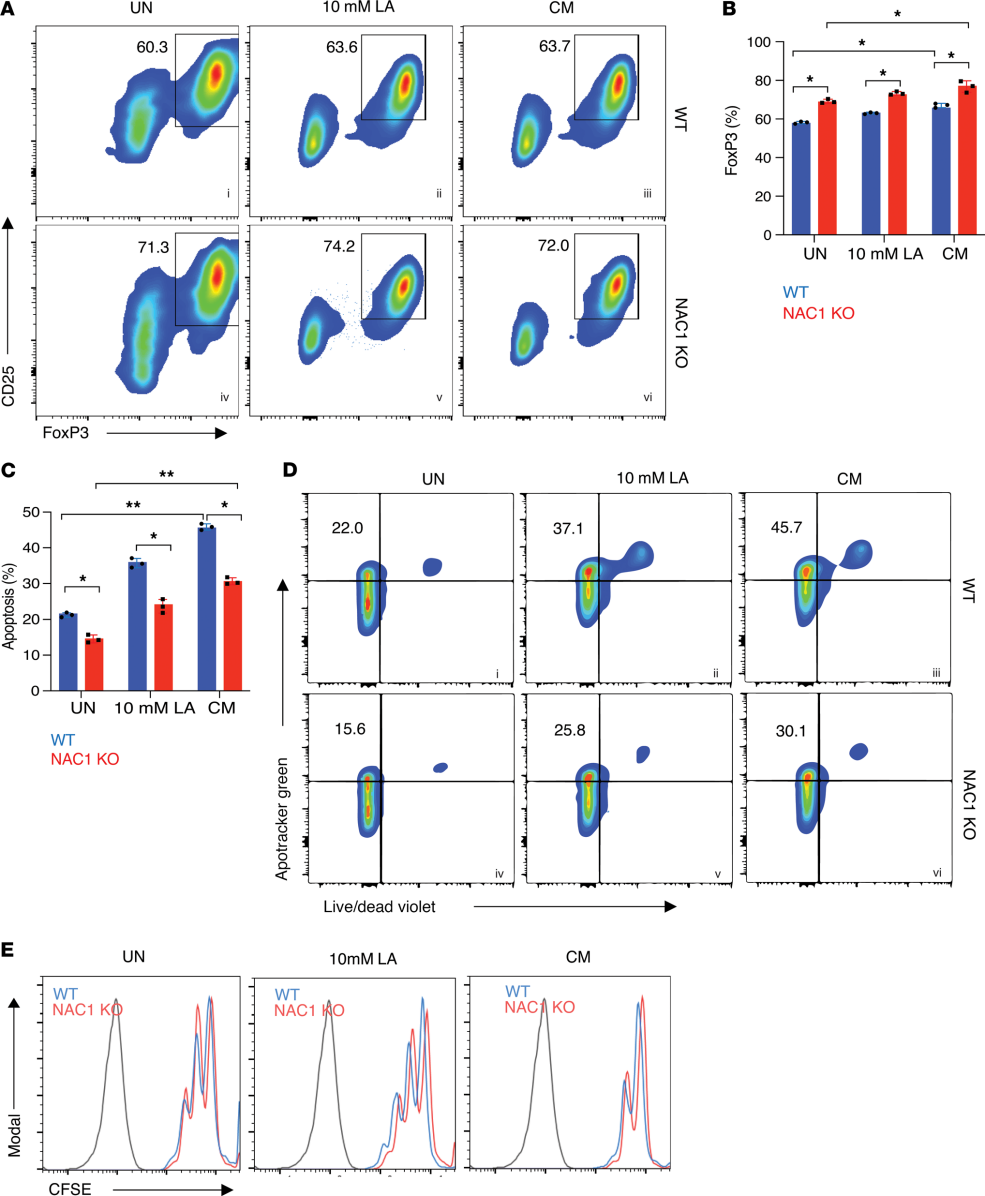
NAC1-KO Tregs show prolonged survival and enhanced polarization in acidic environments. (**A**) Representative flow cytometry shows FoxP3^+^CD25^+^ Treg frequency following LA and CM treatment. (**B**) Quantification of differential expression of FoxP3^+^ Treg frequency after the indicated treatment for 48 hours (*n* = 3). (**C**) Quantification of apoptosis in Tregs after the indicated treatment for 48 hours (*n* = 3). (**D**) Representative plots of Apotracker Green and Live/Dead expression on the WT and NAC1-KO Tregs treated with LA or CM for 48 hours (*n* = 3). (**E**) Proliferation of Tregs as determined by CFSE staining (*n* = 3). The data are represented as mean ± SEM. The differences were analyzed by 2-way ANOVA with multiple comparisons correction using GraphPad Prism. *: *P* ≤ 0.05, **: *P* ≤ 0.01.

To demonstrate the effect of NAC1 on Tregs in a harsh environment, we first compared the survival of NAC1-KO Tregs with that of WT Tregs. CD4^+^CD25^–^ conventional T (Tconv) cells were isolated from FOXP3-IRES-mRFP (FIR) reporter mice, then cultured under polarizing conditions to generate RFP^+^ induced Tregs (iTregs), and the optimal polarization efficiency was confirmed on various days using flow cytometry (Supplemental Figure 1, A and B; supplemental material available online with this article; https://doi.org/10.1172/jci.insight.186000DS1). We also validated a significant difference of FoxP3 expression (Supplemental Figure 1C) in iTregs and similar IL-10 (Supplemental Figure 1D) and TGF-β (Supplemental Figure 1E) secretion profile in natural Tregs and iTregs. Subsequently, we isolated CD4^+^CD25^–^ Tconv cells from both WT and NAC1-KO mice, then cultured them under identical polarizing conditions to generate iTregs. On day 5, WT and NAC1-KO iTregs were cultured in media treated with 10 mM LA or B16-F10 conditioned media (CM) for 48 hours. As NAC1 deficiency promotes FoxP3 expression (10), we assessed FoxP3 expression in these cells. Notably, we observed that the untreated (70.1%), LA-treated (72.2%), and CM-treated (75.1%) NAC1-KO iTregs displayed a higher polarization efficiency than the untreated (57.0%), LA-treated (63.6%), and CM-treated (65.7%) WT iTregs (Figure 2, A and B). This observation aligns with the increased frequency of Tregs in the peripheral tissues of NAC1-KO mice as compared with WT mice (10). We further examined whether expression of NAC1 affects Treg proliferation. Using carboxyfluorescein diacetate succinimidyl ester–labeled (CFSE-labeled) WT and NAC1-KO iTregs, we cultured them in LA-treated and CM-treated media and assessed their proliferation using CFSE dilution assay. Interestingly, we found that the proliferation of both WT and NAC1-KO iTregs was unaffected by LA treatment or CM treatment, suggesting that the higher infiltration of Tregs in NAC1-KO mice is not associated with altered proliferation of Tregs (Figure 2E). We also determined whether NAC1 deficiency has any effect on apoptosis and found that the untreated (15.6%), LA-treated (25.8%), and CM-treated (30.1%) NAC1-KO iTregs exhibited significantly lower levels of apoptosis than the WT iTregs subjected to the same treatment (22.0%, 37.1%, and 45.7%, respectively) (Figure 2, C and D). These results indicate that NAC1-KO iTregs are significantly less apoptotic and more resistant to the stress caused by an acidic environment and suggest that deficiency of NAC1 may promote the survival of Tregs in the TME, thereby increasing infiltration of Tregs to tumors.

### Loss of NAC1 leads to enhanced functional activity of Tregs.

**Figure 3:**
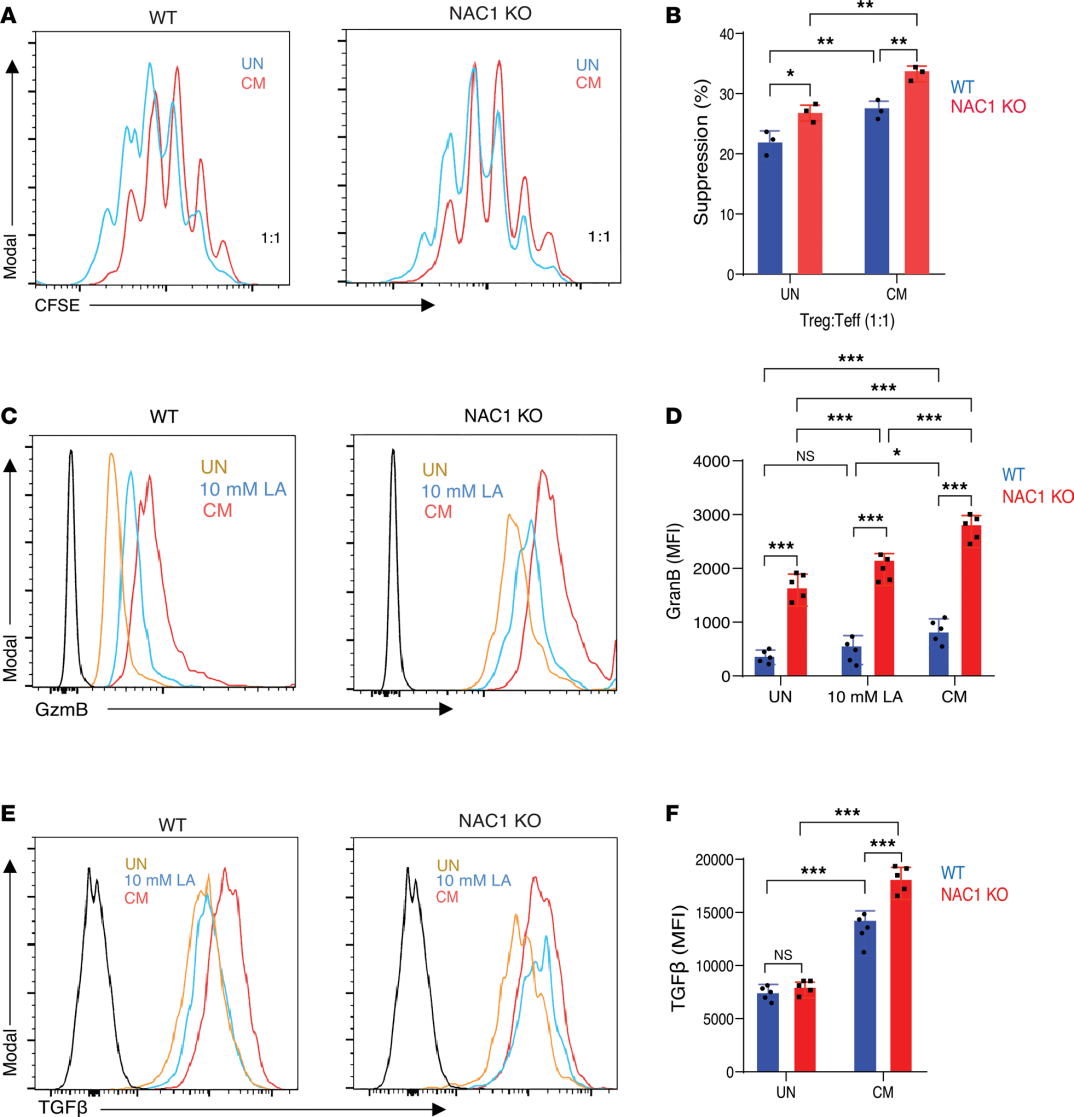
NAC1-KO Tregs show enhanced functional activity of Tregs in acidic environments. (**A**) CD8^+^ cells were labeled with CFSE and cocultured with WT or NAC1-KO Tregs (1:1) in the presence of anti-CD3 and -CD28 antibodies. Histogram of representative experiment showing the proliferation of CD8^+^ cells in the CM-treated culture. (**B**) Quantification analysis of the in vitro suppression assay (*n* = 3). (**C**) Representative histogram of the expression of GzmB in WT and NAC1-KO Tregs after 48-hour treatment with LA or CM (*n* = 5). (**D**) Quantification of differential expression of GzmB in WT versus NAC1-KO Tregs after the indicated treatment for 48 hours (*n* = 5). (**E**) Representative histogram of TGF-β expression in WT Tregs and NAC1-KO Tregs after 48-hour treatment with LA or CM (*n* = 5). (**F**) Quantification of differential expression of TGF-β in WT Tregs versus NAC1-KO Tregs after the indicated treatment for 48 hours (*n* = 5). The data are represented as mean ± SEM. *: *P* ≤ 0.05, **: *P* ≤ 0.01, ***: *P* ≤ 0.001, 2-way ANOVA with multiple comparisons correction using GraphPad Prism.

We next compared the functional activity of NAC1-KO Tregs with that of WT Tregs using an ex vivo suppression assay. In this assay, we stimulated the CFSE-labeled CD8^+^ effector T cells and then cocultured them in control media or CM with FACS-sorted Tregs isolated from the spleen (SPL) and lymph nodes (LNs) of WT or NAC1-KO mice. The suppressive function was assessed by analyzing CFSE dilution using flow cytometry. Our findings revealed that NAC1-KO Tregs were significantly more suppressive compared with WT Tregs in both untreated and CM-treated conditions (Figure 3, A and B, and Supplemental Figure 4A). Further, the expression of Granzyme B (GzmB), an enzyme that is highly expressed in tumor-infiltrating Tregs (18) and plays an important role in increasing the metastatic burden in the lungs and eliminating Tconv cells in colorectal cancer (19, 20), was significantly higher in NAC1-KO iTregs than in WT iTregs in the control media, LA-containing media, or CM (Figure 3, C and D, and Supplemental Figure 3B), suggesting that GzmB is an important mediator of the suppressive capacity of NAC1-KO Tregs. Also, NAC1-KO iTregs produced significantly higher numbers of suppressive cytokine TGF-β than WT iTregs, as evidenced by intracellular staining (Figure 3, E and F). We also conducted bioinformatics analysis in human tumor samples submitted to The Cancer Genome Atlas (TCGA) database, and we found that higher FoxP3^+^ Tregs correlated with lower survival in kidney renal clear cell carcinoma (KIRC) (Supplemental Figure 5, A and B) and glioblastoma multiforme (GBM) (Supplemental Figure 5, C and D). Furthermore, lower expression of NAC1 in intratumoral Tregs also correlated with lower survival (Supplemental Figure 5E). These results indicate that NAC1 has negative effects on the suppressive function of Tregs.

### Loss of NAC1 results in upregulation of CD36 expression and elevation of lipid metabolism of Tregs in acidic environments.

**Figure 4:**
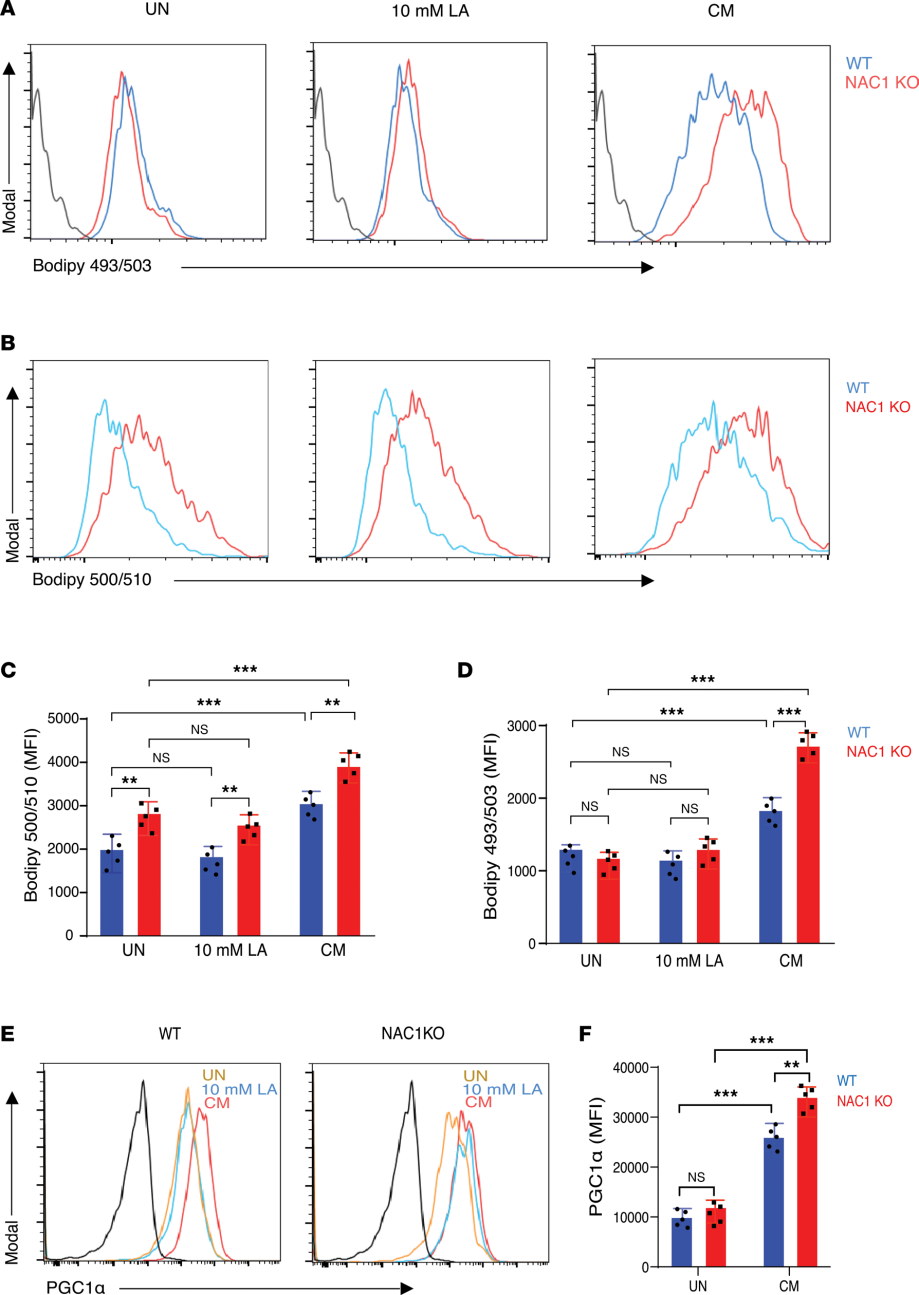
NAC1-KO Tregs have elevated lipid uptake and neutral lipid content in an acidic condition. (**A**) Representative histogram of lipid content measured by BODIPY 493/503 staining in Tregs in the indicated culture conditions. (**B**) Representative histogram of fatty acid uptake measured by C1-BODIPY 500/510 C12 staining in Tregs in the indicated culture conditions. (**C**) Quantification of fatty acid uptake (C1-BODIPY 500/510 C12; *n* = 5). (**D**) Quantification of lipid content (BODIPY 493/503; *n* = 5). (**E**) Representative histogram of PGC-1α expression in WT and NAC1-KO Tregs in the indicated culture conditions. (**F**) Quantification of PGC-1α expression in WT and NAC1-KO Tregs in the indicated culture conditions. The data are represented as mean ± SEM. The differences were analyzed by 2-way ANOVA with multiple comparisons correction using GraphPad Prism software. **: *P* ≤ 0.01, ***: *P* ≤ 0.001.

Since we observed that deletion of NAC1 prolonged the survival and enhanced the function of Tregs in an acidic environment (Figures 2 and 3) and NAC1 has a critical role in metabolic reprogramming (3), we inquired whether the effects of NAC1 on fitness and function of Tregs are mediated through altered metabolism in Tregs. Because CD36, a phagocytic receptor that mediates fatty acid–induced metastasis via regulating fatty acid intake and metabolism, is selectively upregulated in the intratumoral Tregs and functions as a central metabolic modulator that fine-tunes mitochondrial fitness in LA-rich TME (16, 21–23), we then examined the expression of CD36 in the tumor-infiltrating Tregs. Tregs were isolated from the SPL, LNs, and tumors of the B16-F10 melanoma–bearing NAC1-KO mice or WT mice, and the expression of CD36 on WT and NAC1-KO iTregs was analyzed at 24 hours, 48 hours, and 72 hours following treatment with 10 mM of LA. Interestingly, NAC1-KO iTregs exhibited significantly higher CD36 expression than WT iTregs at 48 hours (1.09% vs. 0.43%) and 72 hours (3.79% vs. 1.16%) following the treatment, respectively, though the expression was similar at 24 hours after treatment (Supplemental Figure 2, A and B). Notably, tumor-associated Tregs showed significantly higher expression of CD36 than normal Tregs from the SPL and LNs (Supplemental Figure 2, C and D). These results indicate that the deletion of NAC1 causes an increased CD36 expression under acidic conditions. Because NAC1 has a role in promoting expression of fatty acid synthase (24) and CD36 participates in regulation of lipid metabolism (25), we next determined whether the increased expression of CD36 in NAC1-KO Tregs was associated with altered lipid metabolism. We examined lipid uptake through BODIPY FL C12 (BODIPY 500/510) staining and neutral lipid content through BODIPY (BODIPY 493/503) staining in iTregs treated with LA or CM. Our experiments found a substantially higher level of neutral lipids in NAC1-KO iTregs treated with CM than that in WT iTregs treated with CM, as evidenced by the staining of BODIPY 493/503 (Figure 4A). Similarly, NAC1-KO iTregs internalized a significantly higher amount of BODIPY 500/510 than WT iTregs under all the conditions (untreated, LA-treated, and CM-treated (Figure 4B), with the most pronounced increase observed in the CM-treated cells (Figure 4, C and D). Furthermore, we analyzed the expression of the transcription coactivator PGC-1α (also known as PPARGC1A), which is a master regulator of lipid metabolism, fatty acid transporters (such as FAT/CD36 and FABP3), and mitochondrial biogenesis and coordinates with enhanced OXPHOS and the electron transport chain (26, 27). Our analysis showed that the level of PGC-1α in the CM-treated NAC1-KO iTregs was significantly higher than the corresponding WT iTregs, although both WT and NAC1-KO iTregs exhibited a significantly increased PGC-1α expression when treated with CM (Figure 4, E and F). These results indicate that NAC1 depletion is associated with increased PGC-1α expression, CD36 expression, and enhanced fatty acid transport and metabolism, rendering NAC1-KO Tregs metabolically more active and better adapted to the environmental stress. These results demonstrate the role of NAC1 in modulating Treg metabolism, especially in acidic environments, and imply the metabolic adaptability of NAC1-KO Tregs in an unfavorable TME.

### Loss of NAC1 improves mitochondrial fitness of Tregs.

**Figure 5:**
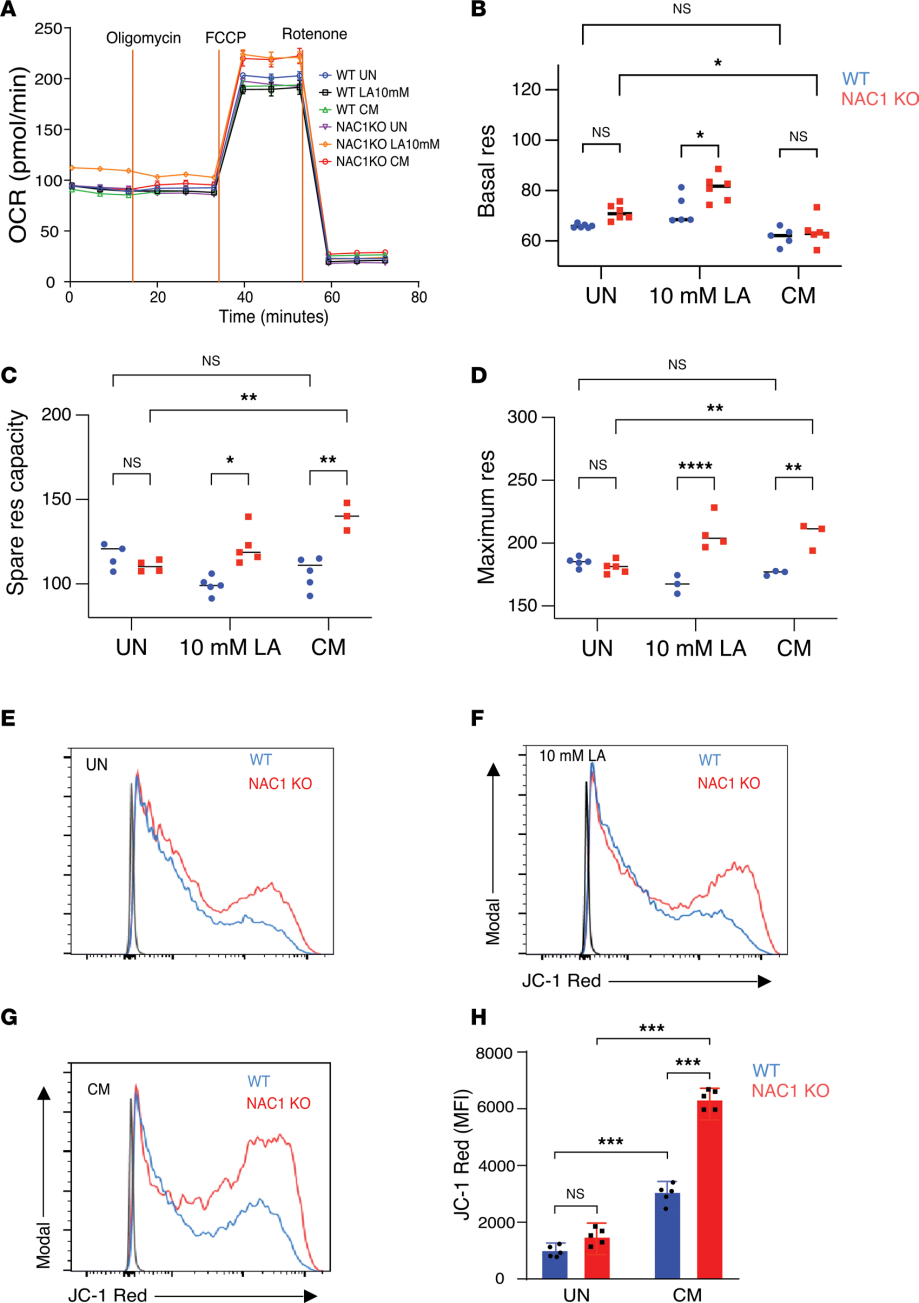
NAC1-KO Tregs show enhanced mitochondrial respiration in an acidic environment. (**A**) Effect of LA or CM treatment on mitochondrial respiration of WT and NAC1-KO Tregs, as measured by Seahorse XFe96 Metabolic Analyzer. Data are represented as mean ± SEM; *n* = 6 per condition from 2 independent experiments. (**B**) Basal respiration. (**C**) Spare respiratory capacity. (**D**) Maximum respiration quantified by Seahorse wave 3.0 software. (**E**–**H**) The mitochondrial membrane potential of WT versus NAC1-KO Tregs measured by JC-1 staining (*x* axis of **E**–**G**). (**E**) Untreated. (**F**) 10 mM LA. (**G**) CM. (**H**) Quantification of mitochondrial membrane potential (*n* = 5). Data are represented as mean ± SEM. The differences were analyzed by 2-way ANOVA with multiple comparisons correction using GraphPad Prism software. *: *P* ≤ 0.05, **: *P* ≤ 0.01, ***: *P* ≤ 0.001.

PGC-1α is a key regulator of mitochondrial biogenesis and coordinates with enhanced oxidative phosphorylation and the electron transport chain, playing a crucial role in cellular energy and metabolism (26, 27). Our observation of the upregulated PGC-1α and FoxP3 in NAC1-KO iTregs prompted us to determine the effects of NAC1 deficiency on energy metabolism and mitochondrial fitness in Tregs following LA or CM treatment. We found that mitochondrial respiration, as indicated by the oxygen consumption rate (OCR) or OXPHOS, was significantly higher in NAC1-KO iTregs following treatment with LA or CM for 48 hours, as analyzed by the mitochondrial stress test using oligomycin, FCCP, and rotenone/antimycin A in a Seahorse bioanalyzer (Figure 5A). We also examined different components of mitochondrial respiration, including basal respiration, ATP-linked respiration, maximal respiration, and spare respiratory capacity, which reflect mitochondrial and cellular fitness (28, 29). We did not observe any significant difference in basal respiration between the untreated NAC1-KO iTregs and WT iTregs (Figure 5B); however, both the spare respiratory capacity (the ability of cells to respond to changes in energetic demand) (Figure 5C) and maximal respiration (reflecting maximum capacity of the electron respiratory chain) (Figure 5D) were significantly higher in NAC1-KO iTregs than that in WT iTregs treated with LA or CM. These results imply that NAC1-KO Tregs are more capable of meeting high energy demands in an acidic environment. Furthermore, we examined the mitochondrial membrane potential of the Tregs using JC-1 staining. We observed that the mitochondrial membrane potential of NAC1-KO iTregs was significantly higher than that of WT iTregs in the presence of LA or CM (Figure 5, E–H). These results provide additional evidence that NAC1 deficiency in Tregs appears to contribute to increased mitochondrial fitness and biogenesis, enabling them to better meet the energy demands within the TME.

### Loss of NAC1 in Tregs facilitates their tumor infiltration and supports tumor progression.

**Figure 6:**
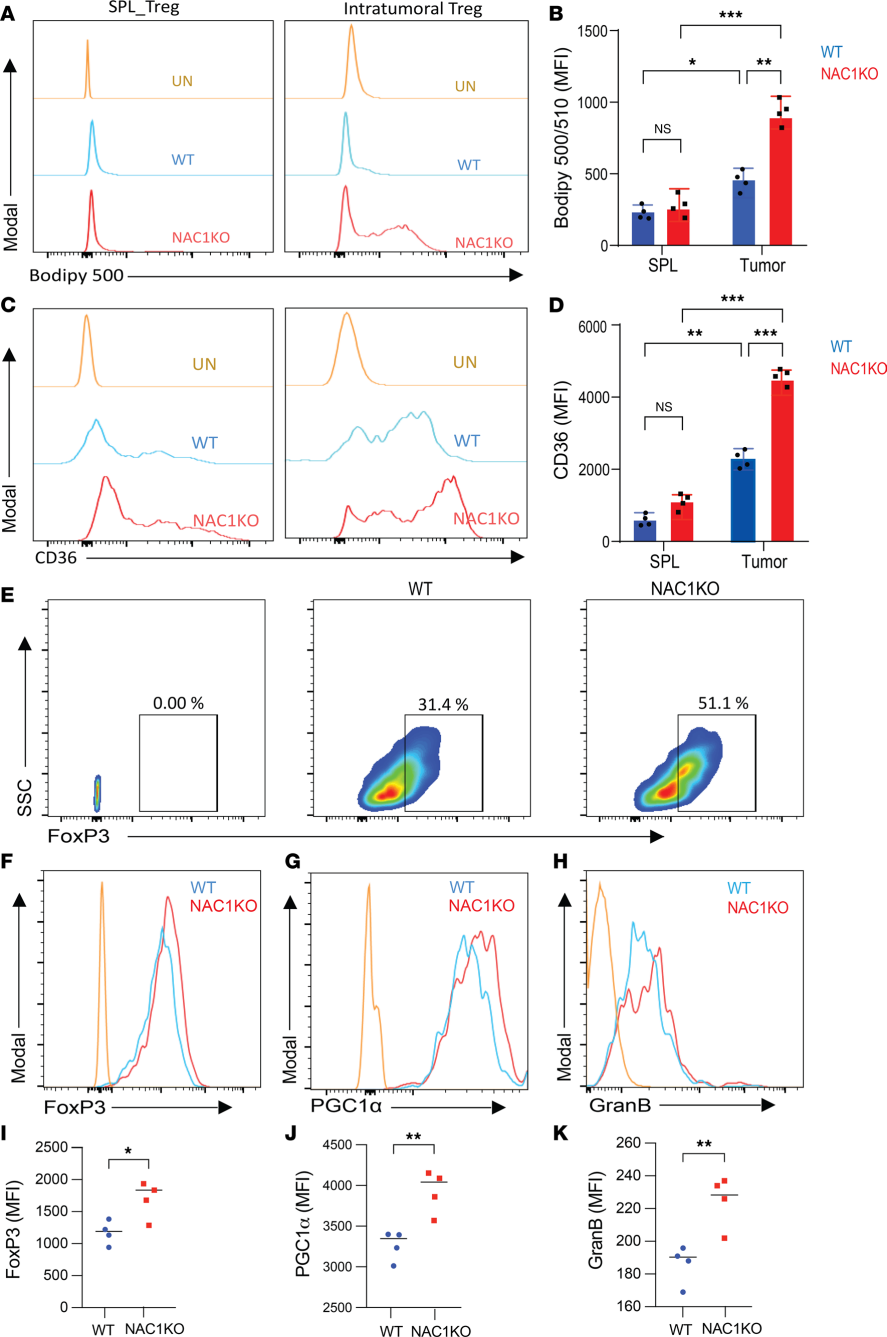
NAC1-KO Tregs are robust in the TME. B16-F10 tumors from WT or NAC1-KO mice were harvested, dissociated, and the tumoral Tregs were stained and analyzed by flow cytometry. (**A** and **B**) Fatty acid uptake of intratumoral Tregs and splenic Tregs as measured by C1-BODIPY 500/510 C12. (**A**) Representative histogram; (**B**) quantification of fatty acid uptake. (**C** and **D**) CD36 expression in intratumoral Tregs and splenic Tregs. (**C**) Representative histogram; (**D**) quantification of CD36 expression (*n* = 3). (**E**) FoxP3^+^CD4^+^ Treg frequency in tumors isolated from WT or NAC1-KO mice. Representative histogram of expression of FoxP3 (**F**), PGC-1α (**G**), and GzmB (**H**) and their quantification: FoxP3 (**I**), PGC-1α (**J**), and GzmB (**K**) (*n* = 4). Data are represented as mean ± SEM. The differences were analyzed by 2-way ANOVA with multiple comparisons correction using GraphPad Prism software. *: *P* ≤ 0.05, **: *P* ≤ 0.01, ***: *P* ≤ 0.001.

Considering the crucial impact of NAC1 deletion on mitochondrial respiration (OXPHOS) and the observed increases in OCR, FoxP3 expression, PGC-1α expression, lipid metabolism, and overall metabolic fitness in NAC1-deficient Tregs in acidic conditions, we further validated our findings by examining CD36 expression, lipid uptake, PGC-1α expression, GzmB expression, and Treg infiltration in B16-F10 tumors isolated from WT and NAC1-KO mice. To assess lipid uptake and CD36 expression, we first digested the tumor tissue using a tumor dissociation kit (Miltenyi Biotec), isolated CD3 cells using a CD3 selection kit (BioLegend), and then stained these isolated CD3 cells with BODIPY 500/510 and CD36. Lipid uptake (BODIPY 500/510) was significantly higher in the intratumoral NAC1-KO Tregs than that in the intratumoral WT Tregs (Figure 6, A and B). Similarly, CD36 expression of the intratumoral NAC1-KO Tregs was significantly higher than that of WT Tregs (Figure 6, C and D). Interestingly, both the intratumoral WT Tregs and NAC1-KO Tregs had significantly higher CD36 expression than the splenic Tregs (Figure 6, C and D). Moreover, the infiltration of Tregs into the tumors was markedly higher in NAC1-KO mice (51.1%) than in WT mice (31.4%) (Figure 6E). This observation was further confirmed by immunostaining of tumor tissue sections, which revealed a higher infiltration of CD36-expressing Tregs in NAC1-KO mice (Figure 1E). Additionally, NAC1-KO intratumoral Tregs displayed higher expression of FoxP3 (Figure 6, F and I), GzmB (Figure 6, H and K), and PGC-1α (Figure 6, G and J) compared with WT intratumoral Tregs. These results verified that NAC1 deletion in Tregs led to enhanced lipid metabolism and mitochondrial biogenesis. Additionally, the enhanced infiltration and suppressive effects of NAC1-deficient Tregs in the TME suggest that NAC1 deficiency in Tregs may contribute to faster tumor initiation and growth in NAC1-KO mice compared with WT mice.

### Specific depletion of NAC1 in Tregs is a critical factor that supports tumor progression.

**Figure 7:**
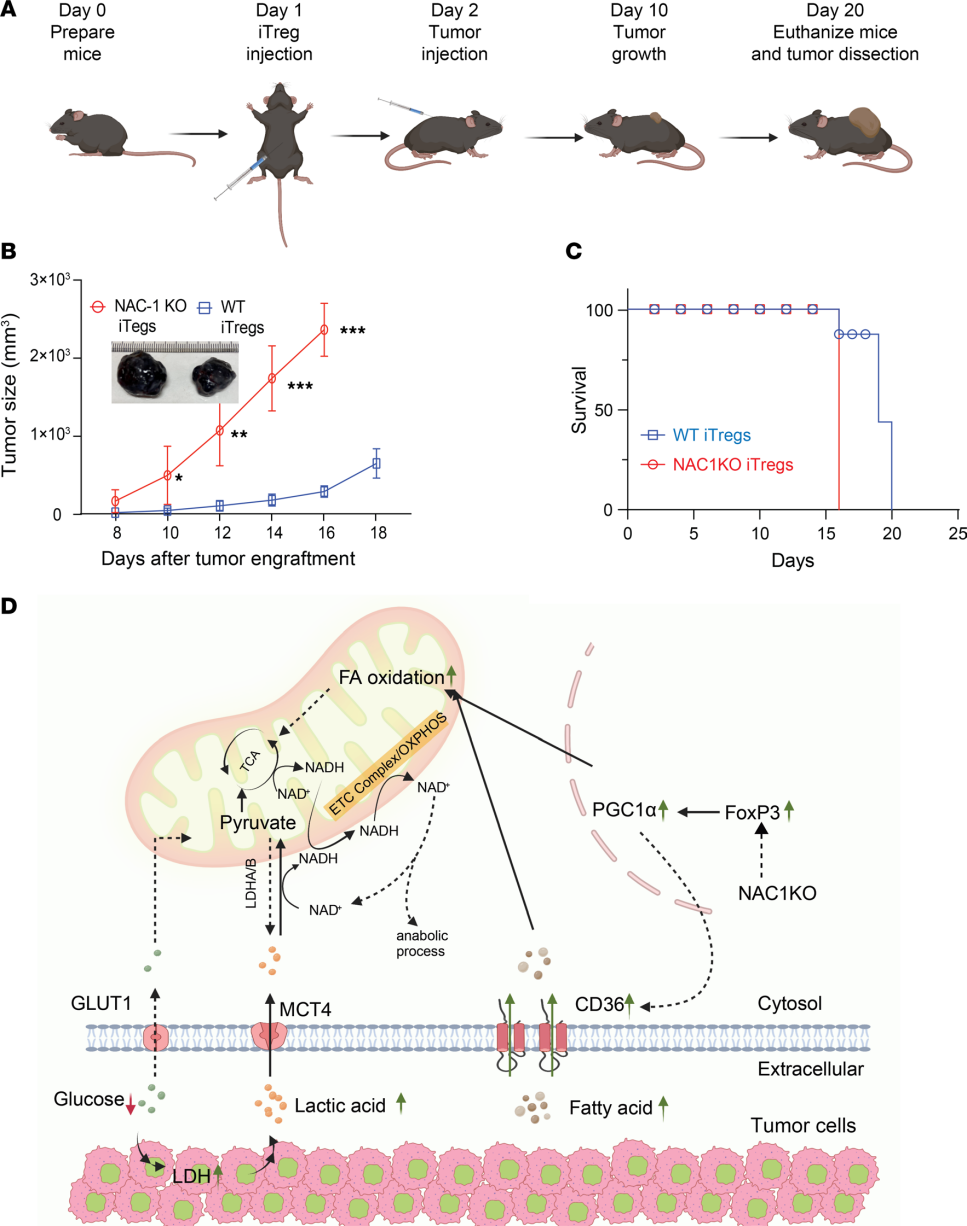
NAC1-KO Tregs support tumor growth. Three million Tregs were i.p. injected in each of the recipient Thy1.1 congenic mice, followed by tumor engraftment. (**A**) Schematic representation of adoptive Treg transfer and tumor engraftment. (**B**) Tumor growth curve of B16-F10 melanoma (*n* = 5). (**C**) Representative survival curve. (**D**) Illustration of the proposed and the rational pathway regulated by NAC1-FoxP3, leading to mitochondrial fitness in the TME. *: *P* ≤ 0.05, **: *P* ≤ 0.01, ***: *P* ≤ 0.001, unpaired *t* test.

To further substantiate the role NAC1-deficient Tregs and their suppressive function in tumor progression, we conducted an adoptive transfer experiment in which the WT iTregs or NAC1-KO iTregs (Thy1.2^+^) were transferred into Thy1.1^+^ congenic recipient mice on day 1, and on the following day, B16-F10 melanoma cells were subcutaneously injected into the flank of the recipient mice (Figure 7A). Remarkably, the tumors in Thy1.1^+^ congenic recipient mice that received NAC1-KO iTregs grew significantly faster than the tumors in mice that received WT iTregs (Figure 7B), leading to shorter survival (Figure 7C). Similarly, MC38 tumors also exhibited significantly faster growth in mice receiving NAC1-KO iTregs than tumors in mice receiving WT iTregs (Supplemental Figure 3A). In another experiment, WT iTregs or NAC1-KO iTregs (Thy1.2^+^) were adoptively transferred into Thy1.1^+^ congenic recipient mice following B16-F10 tumor engraftment. The results demonstrated that NAC1-KO iTregs promoted tumor growth (Supplemental Figure 7). These experiments demonstrate that loss of NAC1 in Tregs causes their metabolic reprogramming and enhances robustness of mitochondria in an acidic TME, leading to increases of Treg infiltration. The enhanced PGC-1α expression leads to increased lipid metabolism, and mitochondrial fitness within the TME, contributing to tumor progression (Figure 7D).

## Discussion

NAC1 promotes glycolysis and the survival of hypoxic tumor cells, possibly through the direct regulation of c-Myc. Deletion of NAC1 in tumor cells leads to oxidative stress, reduced LDHA activity, and enhanced infiltration of CTLs within the tumor mass (8). Compared with Tconv cells, Tregs have a significantly reduced NAC1 expression. Deletion of NAC1 results in increased acetylation of FoxP3, leading to enhanced FoxP3 expression and the suppressive function of Tregs. NAC1 deletion was found to impair T cell development in the thymus. However, in peripheral blood and secondary lymphoid organs, Treg function is primarily regulated by FoxP3, which is upregulated in the absence of NAC1. These findings suggest that while NAC1 influences broader aspects of T cell biology, its effects on Tregs are largely FoxP3 dependent (10). Additionally, the critical role of NAC1 in memory T cell development has been recently reported (4, 9). Prompted by our recent findings that NAC1 is a critical suppressor of Treg development and function, and this role of NAC1 is mediated through epigenetic regulation of FoxP3 expression and Treg stability (10), in the current study, we investigated the implications of the NAC1-mediated control of Tregs in tumor progression. We show here that Tregs with deficiency in NAC1 have enhanced metabolic capacity to adapt to the acidic TME, primarily through CD36/PGC-1α–driven enhancement of mitochondrial fitness and lipid metabolism, and NAC1-deficient Tregs promote tumor growth by increasing their tumor infiltration and strengthening their suppressive function within the TME (Figure 7D).

Our analysis of Tregs from WT and NAC1-KO mice found that in the LA-containing media or CM, the survival of NAC1-KO Tregs is prolonged as compared with that of WT Tregs (Figure 2, C and D), suggesting that NAC1 deficiency confers a survival advantage to Tregs, allowing them to thrive in the TME. This is likely due to the upregulation of FoxP3 expression in NAC1-KO Tregs, which reprograms T cell metabolism by suppressing glycolysis but promoting OXPHOS, rendering Tregs resistant to lactate inhibition. The increased NAD/NADH ratio in Tregs, driven by FoxP3-mediated metabolic changes, may enable them to effectively utilize lactate and convert it into pyruvate and favor Tregs to survive in the TME, where lactate is abundant. Indeed, we show that lipid metabolism and mitochondria activity are enhanced in Tregs deficient in NAC1 when cultured in the LA-rich media or CM (Figure 3). We also found that loss of NAC1 in Tregs results in a significant increase in CD36 expression, a key fatty acid receptor, in Tregs cultured in CM (Supplemental Figure 2) or on the tumoral Tregs (Figure 6). Selective upregulation of CD36 in intratumoral Tregs and its role as a central metabolic modulator that fine-tunes mitochondrial fitness in the context of LA-rich TME were reported previously (16, 21–23). This upregulation was accompanied by an increased fatty acid uptake, as evidenced by BODIPY 500/510 staining (Figure 4A), and by an increase in neutral fat content, as indicated by BODIPY 493/503 staining, particularly in CM-treated or intratumoral NAC1-KO Tregs (Figure 6, A and B). Consistent with these findings, we also observed the upregulation of PGC-1α, a critical regulator of mitochondrial biogenesis in NAC1-KO Tregs. In Seahorse analysis, we found that NAC1-KO Tregs display significantly higher levels of maximal respiration and spare respiratory capacity when cultured in CM (Figure 5). These observations imply that depletion of NAC1 leads to upregulation of expression of PGC-1α, CD36, and fatty acid transport and metabolism, collectively endowing NAC1-KO Tregs with increased metabolic vigor and adaptability within the acidic TME.

Furthermore, NAC1-KO Tregs show a significant enhancement in their suppressive function, as demonstrated by in vitro suppression assays (Figure 3) and evidenced by a substantial increase in GzmB and TGF-β in NAC1-KO Tregs, particularly when cultured in CM (Figure 3, C and D) or in the tumoral NAC1-KO Tregs (Figure 3, E and F), implying that NAC1-deficient Tregs possess higher suppressive activity over CTLs within the acidic TME. These results may explain our in vivo experiments showing that absence of NAC1 in Tregs supports tumor growth in B16 melanoma (Figure 7 and Supplemental Figure 7) and MC38 colon carcinoma models (Supplemental Figure 3A). Additionally, IMC analysis of the tumor tissue sections shows that the increased tumor infiltration of Tregs is associated with an increase in apoptotic CD8^+^ cells expressing the PD-1 marker (CD8^+^PD-1^+^) (Figure 1, F and G). Together, these experiments pinpoint the role of NAC1 in controlling the survival, metabolic fitness, and suppressive function of Tregs; all are causally associated with immune evasion in the TME (14–17).

It is worth noting that the mice employed in this study were subjected to complete knockout of the *NAC1* gene. Therefore, the deficiency of NAC1 in other types of cells may also affect tumor progression. In particular, the precise roles of NAC1 in other T cell subtypes, including CD8^+^ T cells and conventional CD4^+^ T cells (e.g., Th1, Th2, T follicular helper, and Th17), and in innate immune cells, like macrophages and dendritic cells, are relatively unexplored. Thus, the exact impact of NAC1 on tumor development and progression remains to be further delineated.

## Methods

### Sex as a biological variable.

Sex was not considered as a biological variable; both female and male mice were used.

### Cell lines and mice.

The B16-F10 (ATCC CRL-6475) melanoma cell line and MC38 colon adenocarcinoma cell line (gift from James W. Hodge, National Cancer Institute, National Institutes of Health, Bethesda, Maryland, USA) was cultured in Dulbecco’s modified Eagle medium with 10% fetal bovine serum (FBS) and 1% penicillin-streptomycin and used for experiments when in the exponential growth phase. All reagents were from MilliporeSigma. C57BL/6 (B6), FIR reporter, and Thy1.1^+^ congenic mice were purchased from The Jackson Laboratory. NAC1-KO mice were generated by Jianlong Wang, Columbia University Irving Medical Center, and crossed in the B6 background for more 10 generations (10). All the animal experiments were performed in compliance with the regulations of The Texas A&M University Animal Care Committee (IACUC no. 2018-0065) and in accordance with the guidelines of the Association for the Assessment and Accreditation of Laboratory Animal Care.

### T cell culture and proliferation/cell division.

WT and NAC1-KO T cells were isolated from mice using T cell isolation kits including mouse CD4^+^ (no. 130-104-454), CD8a^+^ (no. 130-104-075), and CD4^+^CD25^+^ Tregs (no. 130-091-041); were activated by anti-mouse CD3 antibody (clone 2C11; BioLegend) plus anti-mouse CD28 antibody (clone 37.51; BioLegend) in RPMI 1640 media (Biological Industries) with 10% FBS (Omega Scientific); and were monitored for their survival by trypan blue cell exclusion method using a TC20 automated cell counter (Bio-Rad). In vitro T cell survival was determined using trypan blue exclusion. Proliferation/division of T cells was measured using the CellTrace CFSE Cell Proliferation Kit (no. C34554, Invitrogen).

### Cancer cell–conditioned medium and iTreg culture.

iTregs were generated by activating naive CD4^+^ T cells with anti-CD3 (clone 145-2C11) and anti-CD28 (clone 37.51) monoclonal antibodies (BioLegend) in RPMI 1640 media supplemented with 10% FBS, 5 ng/mL TGF-β, and 5 ng/mL IL-2 (recombinant) for 3 days. Then, activated CD4^+^ T cells were maintained in RPMI media plus 10% FBS and 10 ng/mL IL-2 for another 2 days. Differentiated iTregs were first sorted using a FACS cell sorter (FACSAria III sorter, BD Biosciences) and then incubated in cancer cell–conditioned medium and under the indicated culture conditions for 48 hours. Control RPMI 1640 for the treatment of iTregs in vitro was prepared with RPMI 1640 supplemented with 2 mM glucose, 10 mM glutamine, 10% dialyzed FBS, 0.1% β-mercaptoethanol, and the indicated concentrations of LAs as we previously described (8). B16-F10 cancer cell–conditioned medium was collected by incubating B16-F10 cells (70%–80% confluent) with the control RPMI 1640 described above for 18 hours. Then, the culture medium was collected and centrifuged at 200*g* for 15 minutes to remove debris and cancer cells as cancer cell–conditioned medium. B16-F10 cancer cell–conditioned medium collected as described above was passed through a 0.2 μm membrane filter (MilliporeSigma) before Treg culture at a volume ratio of 1:3.

### In vitro mouse Treg generation.

CD4^+^CD25^–^ naive T cells sorted from SPL and LNs of WT or NAC1-KO or FIR mice from CD4^+^ cells were enriched using a negative selection kit (MojoSort Mouse naive CD4 T Cell Isolation Kit; BioLegend). iTregs were generated by activating naive CD4^+^ T cells isolated from SPL and LNs of WT or NAC1-KO mice with anti-CD3 plus anti-CD28 monoclonal antibodies (BioLegend) in RPMI 1640 media supplemented with 10% FBS, 5 ng/mL TGF-β, and 5 ng/mL recombinant IL-2 for 3 days. Then, activated CD4^+^ T cells were maintained in RPMI 1640 media plus 10% FBS and 10 ng/mL IL-2 for another 48 hours. Efficiency of iTreg differentiation was determined by FACS analysis.

### Flow cytometry, cell sorting, and antibodies.

Single-cell suspensions were incubated with TruStain FcX (anti-mouse CD16/32) antibodies (BioLegend) on ice for 10 minutes before staining. Cell suspensions were first stained using a LIVE/DEAD Fixable Violet Dead Cell Stain Kit (Thermo Fisher Scientific) or Zombie NIR Fixable Viability Kit (BioLegend) or Zombie Aqua Fixable Viability Kit (BioLegend) at 37°C for 10 minutes. After washing, surface markers were stained for 30 minutes at 4°C. Intracellular staining was performed after incubation of single-cell suspensions with GolgiStop from BD Biosciences (AB_2869012) in medium for 4 hours using Intracellular Staining Permeabilization Wash Buffer and Fixation Buffer from BioLegend (catalog 421002). Apoptosis was determined by staining with Apotracker Green (BioLegend catalog 427402). Samples were analyzed on Fortessa X-20 flow cytometers (BD Biosciences), and data were analyzed with FlowJo with the gating strategy shown in Supplemental Figure 6. Cells were sorted on a FACSAria III sorter. Tregs were defined by the following staining: Live/Dead^–^CD45^–^CD3^+^CD4^+^CD8^–^CD25^+^FoxP3^+^. CD8^+^ T cells were defined by the following staining: CD45^+^CD3^+^CD8^+^CD4^−^. The following antibodies against mouse proteins were used: anti-CD45 (BioLegend clone 30-F11), anti-CD3ε (eBioscience clone 17A2), anti-CD4 (eBioscience clone RM4-5), anti-CD8α (eBioscience clone 53.6.7), anti-CD44 (eBioscience clone IM7), anti-CD4 (eBioscience clone GK1.5), anti-CD25 (BioLegend clone 3C7), anti-CD36 (BioLegend clone HM36), anti-FoxP3 (BioLegend clone MF-14), anti-GFP/YFP (BioLegend clone FM264G), anti-Thy1.2 (BioLegend clone 30-H12), anti–PGC-1α (Novus Biologicals, Bio-Techne; clone NBP1-04676, polyclonal), and anti–Granzyme B (BioLegend clone GB11).

### Tumor engraftment and murine melanoma models.

Before tumor induction, 8- to 10-week-old mice were shaved on the back on the skin surface, to induce tumor formation. For tumor engraftment, 3 × 10^6^ B16-F10 or 3 × 10^6^ MC38 tumor cells in 100 μL PBS were injected subcutaneously (s.c.) into the right flank of B6.Thy1.1 or WT or NAC1-KO mice. Tumors were measured every 2–3 days after tumor engraftment and calculated. Tumor volumes were calculated by volume = (length × width^2^)/2 for engrafted tumors or volume = (length × width × height) for inducible tumors. When tumors reached a maximum size of 2,000 mm^3^, tumors were prepared for analysis. All experiments were conducted according to Swiss federal regulations.

### Tumor digestion and cell isolation.

Tumors were washed with PBS and minced into small pieces in RPMI containing the enzyme mix from a mouse tumor dissociation kit (Miltenyi Biotec; 130-096-730). Tumor digestion was done according to the manufacturer protocol. Single-cell suspension was filtered through a 100 μm cell strainer (BD Falcon) and washed with PBS. Tumor cells were incubated with RBC Lysing Buffer (BD Biosciences) to lyse red blood cells and then washed with FACS buffer (PBS with 2% FBS and 2 mM EDTA). Tumor-infiltrating T cells were further enriched by a CD3 cell selection kit (BioLegend) as described by the manufacturer. Cells were further stained and analyzed by FACS analysis.

### IMC analysis.

IMC uses heavy metal label–conjugated antibodies, greatly enhancing the deep immunophenotyping analysis of tumor samples. A dimensionality reduction technique, t-SNE, was used to analyze several tumor-associated immune cell markers in the WT versus NAC1-KO groups of mice. A bar graph of the number of cells per neighborhood across the imaged tumor samples was constructed to analyze the local cell densities within individual neighborhoods as described previously (30). Ir191, Er167, Dy162, Er170Sm149, and Yb176 were used for staining DNA, Ki-67 Ag, CD8^+^ T cells, B220 (B cells), CD11b (dendritic cells), and F4/80 (macrophages), respectively.

### Mitochondrial membrane potential, fatty acid uptake, and lipid content assay.

To measure mitochondrial membrane potential, cells were washed and incubated with prewarmed (37°C) staining solution (Dulbecco’s PBS with 1% FBS) and stained with JC-1 assay kit (M34152; Thermo Fisher Scientific) at working concentrations of 1 μM for 30 minutes as suggested by the manufacturer. After staining, the cells were washed and resuspended in fresh FACS buffer for surface marker staining, as described above. To measure fatty acid uptake, cells were incubated in RPMI 1640 medium (or human T cell culture medium) containing C1-BODIPY 500/510 C12 (Life Technologies) at a final concentration of 0.5 μM for 15 minutes at 37°C. After incubation, cells were washed with FACS buffer for surface staining. For lipid content detection, after permeabilization and fixation, cells were stained using BODIPY 493/503 (Life Technologies) at a final concentration of 500 ng/mL together with other intracellular markers.

### Adoptive T cell transfer.

Naive CD4^+^ T cells were harvested using a combination of negative magnetic selection (MojoSort Mouse CD4 T Cell Isolation Kit; BioLegend) and FACS (>98% purity) from SPL and LNs of WT or NAC1-KO mice and cultured in a Treg-polarizing condition as mentioned above. On day 0, approximately 3 × 10^6^ WT and NAC1-KO iTregs (day 5) were i.p. injected into 2 separate groups of Thy1.1 mice (*n* = 5) prepared for tumor engraftment. On day 1, 3 × 10^6^ B16-F10 melanoma or MC32 CEA tumor cells were s.c. injected into each of the recipient mice of both groups and tumor progression was monitored. The mice were monitored for survival and tumor size up to day 28 after tumor induction. The experiment was terminated on day 28, and the explanted tumor was analyzed by flow cytometry, IMC, and confocal microscopy (Olympus FV3000) as described (30).

### Tumor imaging and immunohistochemistry.

Tumor tissues were fixed with 10% neutral formalin solution (VWR) for 24 hours and placed in labeled cassettes for further embedding in molten paraffin wax. Tissue sections of 3–5 µm were prepared from fixed tissue embedded in paraffin block and stained with H&E. For immunofluorescence microscopy, the tissues were frozen in cryovials on dry ice immediately following resection. Cryosectioning and immunofluorescence staining were performed as described (30). Alexa Fluor 488–CD36 (Abcam ab252922), Alexa Fluor 647–FoxP3 (BioLegend 126402), and eFluor 570–Thy1.1 (Thermo Fisher Scientific 14-0900-81) were used to detect the tumor-infiltrating Tregs.

### Ex vivo Treg suppression assay.

CD8^+^ T cells from the SPL and LNs of Thy1.1 mice were enriched using a negative selection kit (MojoSort Mouse CD8 T Cell Isolation Kit; BioLegend) and stained with a CellTrace CFSE Cell Proliferation Kit (Thermo Fisher Scientific) for 10 minutes at room temperature. A total of 2.5 × 10^4^ CD8^+^ cells were seeded into a CD3-coated (4 μg/mL), 96-well, round plate in RPMI 1640 medium containing 4 μg/mL CD28. CD4^+^CD25^+^ Tregs were sorted from SPL and LNs of WT and NAC1-KO mice. CD4^+^ T cells enriched using a negative selection kit (MojoSort Mouse CD4 T Cell Isolation Kit; BioLegend) were added according to the indicated ratios for Tregs to effector T cells. Then 5 ng/mL recombinant IL-2 was supplemented into culture media. As negative controls, CD4^+^CD25^+^ Tregs and CD4^+^CD25^−^ responder T cells were cultured without any stimulus. Cells were incubated at 37°C under 5% CO_2_ for 72 hours, and then the proliferation of CD8^+^ T cells was determined by CFSE dilution with flow cytometry analysis. Suppression of responder T cells was determined.

### Metabolic profiling: Seahorse assay.

Seahorse XFe96 Extracellular Flux Analyzer with Seahorse XF Cell mito stress test kit (103010-100; Agilent) were used according to the user guides provided with the kit. Approximately 2 × 10^5^ iTregs were plated in the Cell-Tak–coated Seahorse Bioanalyzer XFe96 culture plates in assay medium consisting of minimal RPMI 1640 medium supplemented with 1% bovine serum albumin and 25 mM glucose, 2 mM glutamine, and 1 mM sodium pyruvate. Basal rates were taken for 30 minutes; a set of drugs, oligomycin (2 mM), FCCP (0.5 mM), and rotenone/antimycin A (0.5 mM), were injected into each sample at different times; and readings were measured at every 3 minutes for 1 to 2 hours. Each condition was analyzed, with 6–12 replicates in each single experiment. The extracellular acidification rate was measured in the glycolytic rate, and the OCR was tested to indicate OXPHOS.

### TCGA bioinformatics analysis.

The bioinformatic analysis was conducted using data collected from TCGA database, accessed through the GEPIA2 web server (http://gepia2.cancer-pku.cn/). Two specific TCGA subtypes were analyzed: KIRC and GBM. The focus of the analysis was on the expression of Tregs, annotated by the markers FOXP3, CTLA4, CCR8, and TNFRSF9. These curves were generated to compare high- versus low-expression groups for each marker, providing insights into the prognostic significance of Treg expression in KIRC and GBM. The overall survival and disease-free survival rates associated with the expression levels of these Treg markers were illustrated using Kaplan-Meier survival curves. Statistical analyses were performed to assess the significance of survival differences between the high- and low-expression groups. The log-rank test was employed to determine the *P* values, and a *P* value of less than 0.05 was considered statistically significant.

### Statistics.

Paired or unpaired *t* test or 1-way or 2-way ANOVA was performed to analyze the differences between the groups, using GraphPad Prism. For mouse survival curve analysis, the Kaplan-Meier method was applied and compared statistically using the log-rank test in GraphPad Prism. Each point represented a biological replicate, and all data are presented as means ± SD or means ± SEM, as indicated. *P* values are labeled in the figures. *P* < 0.05 was considered statistically significant.

### Data availability.

The datasets shown in all the figures are listed in an associated spreadsheet of Supporting Data Values. All data supporting the findings of this study are available in the main text and its supplement.

## Author contributions

AK and JS conceptualized the research project. AK designed and performed the experiments, analyzed the data, and wrote the manuscript. XX did breeding maintenance of animals used in this study and monitored mice. JKD, HYP, LW, DJB, XR, and YR assisted in data analysis and revised the manuscript. JMY, PDF, and JS supervised the overall project and reviewed, advised on, and revised the manuscript. JMY and JS are the guarantors for the overall content of this work. All authors read and approved the final version of the manuscript. JMY and JS are the guarantors of this work and, as such, had full access to all the data in the study and take responsibility for the integrity of the data and the accuracy of the data analysis.

## Supplementary Material

Supplemental data

Supporting data values

## Version 1 01/07/2025

In-Press Preview

## Version 2 02/24/2025

Electronic publication
