# Supplementary material for: Metabolic fitness of NAC1-deficient Tregs in the tumor microenvironment fuels tumor growth
Source: JCI Insight. 2025 Jan 7;10(4):e186000. doi: 10.1172/jci.insight.186000 (PMC11949012; doi:10.1172/jci.insight.186000)

Supplemental Figure 1. Induction of Treg and characterization: $CD4^+$ T cells were isolated from the spleen and LNs of the FIR reporter mice using a negative selection kit and activated under Tregs polarizing condition. Treg polarization was monitored by flow cytometry analysis. (**A**) Schematic illustration of $CD4^+$ T polarization into Tregs. (**B**) Representative flow cytometry profiles showing the frequency of $CD4^+$ T cell polarized to $FoxP3^+$ Tregs on day 0, day 2, day 4 and day 6. (**C**) FoxP3 (MFI) expression level in WT *vs.* NAC1 KO iTregs. (**D**) IL10 (MFI) expression level WT *vs.* NAC1 KO nTregs and iTregs. (**E**) TGF-β (MFI) expression level WT *vs.* NAC1 KO nTregs and iTregs (n=5).

Supplemental Figure 2. NAC1 KO Tregs show upregulated CD36 expression in the LA-rich culture condition. (**A**) Representative plot of CD36 expression on WT and NAC1 KO Tregs cultured in the presence of 10 mM of LA for 24 hours, 48 hours and 72 hours. (**B**) Quantification of CD36 expression on WT *vs.* NAC1 KO Tregs after treatment with 10 mM LA for 24 hours, 48 hours and 72 hours. (n=5). (**C**) Quantification of CD36 expression in Tregs isolated from the draining lymph node (DLN), spleen (SPL) and tumor from B16 tumor-bearing mice (n=3). (**D**) Representative histogram of CD36 expression in Tregs isolated from the DLN, SPL and tumor from B16 tumor-bearing mice.

Supplemental Figure 3. (**A**) Representative tumor growth curve of MC38 tumor growth after WT (n=5) and NAC1 KO (n=5) iTreg adoptively transferred in Thy1.1 congenic mice. (**B**) Representative histogram showing the expression of GrazB in WT *vs*. NAC1 KO iTreg after 48-hour treatment of LA and CM (n=3).

Supplemental Figure 4. NAC1 KO Tregs show enhanced suppressive activity in acidic environments. (**A**) CD8 cells were labelled with CFSE and co-cultured with WT or NAC1 KO Tregs (1:1) in the presence of anti-CD3 and CD28 antibodies. Histogram of representative experiment showing the proliferation of CD8 cells in the CM-treated culture.

Supplemental Figure 5. Bioinformatics analysis of human samples. The survival curves were generated to compare high versus low expression groups for each marker, providing insights into the prognostic significance of Treg expression in KIRC and GBM. **(A)** Overall survival and **(B)** Disease free survival of

Kidney Renal Clear Cell Carcinoma (KIRC) patients. **(C)** Overall survival and **(D)** Disease free survival of Glioblastoma Multiforme (GBM) patients shows lower survival in patients with higher expression of FoxP3 in intra-tumoral Tregs. **(E)** Lower NAC1expression in intra-tumoral Tregs shows lower survival in patients.

Supplemental Figure 6. Gating strategy for flow cytometry analysis: Lymphocyte were gated (SSC-A vs FSC-A). Doublets were removed from lymphocytes using FSC-A and FSC-H. Dead cells were removed from the analysis using Live/Dead® Aqua fixable stain. Live Lymphocyte gate was further analyzed for CD4 population, and then CD4 cells selected for further characterization of $CD25^+$ $FoxP3^+$ Tregs.

Supplemental Figure 7. NAC1 KO Tregs support tumor growth. Three million Tregs were *i.p.* injected in each of the recipient $Thy1.1^+$ congenic mice after tumor engraftment. (**A**) Schematic representation of adoptive tumor engraftment and Treg transfer. (**B**) Tumor growth curve of B16F10 melanoma (n=5). (**C**) Representative survival curve.

Supplemental Figure 1

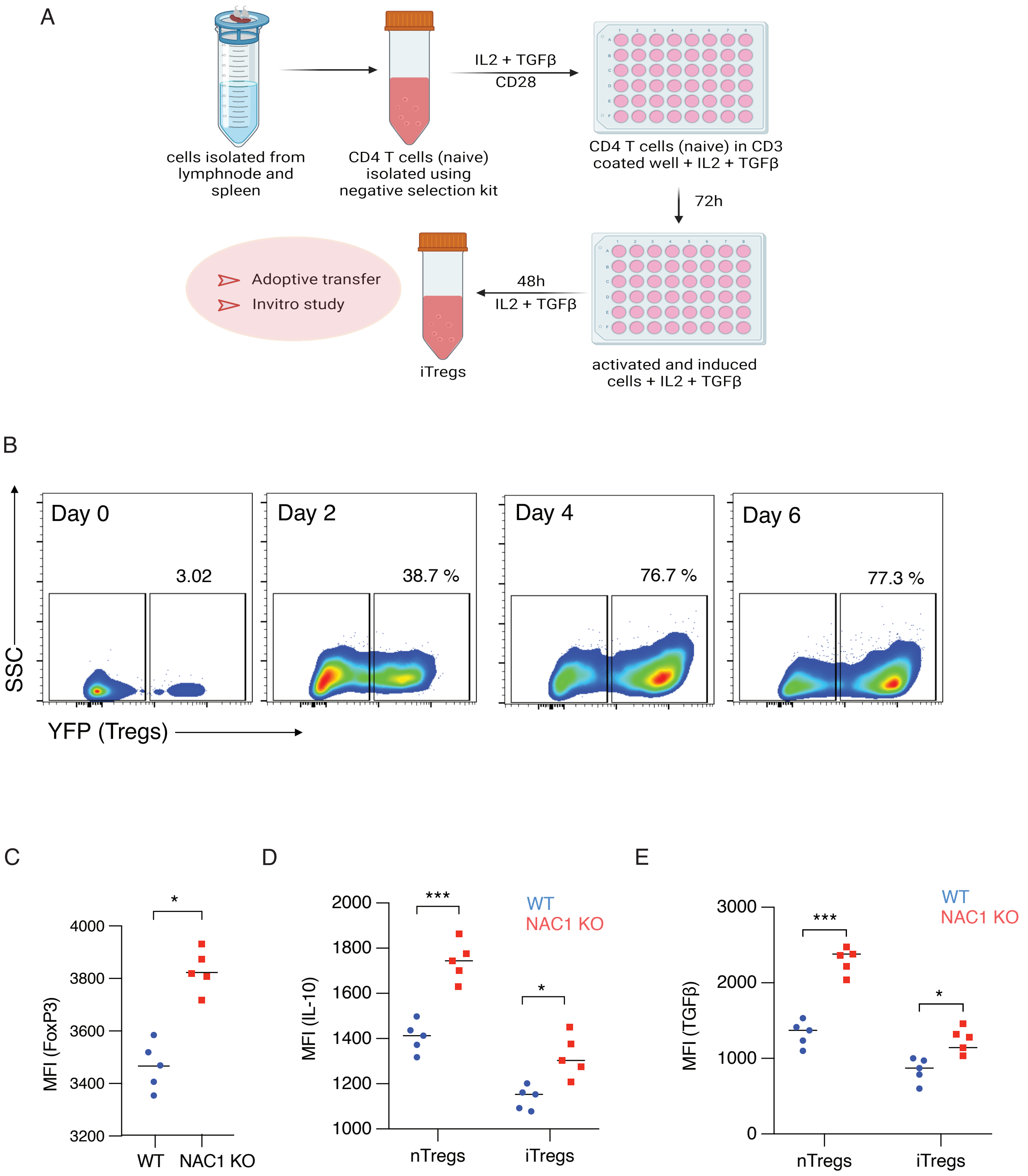

Supplemental Figure 2

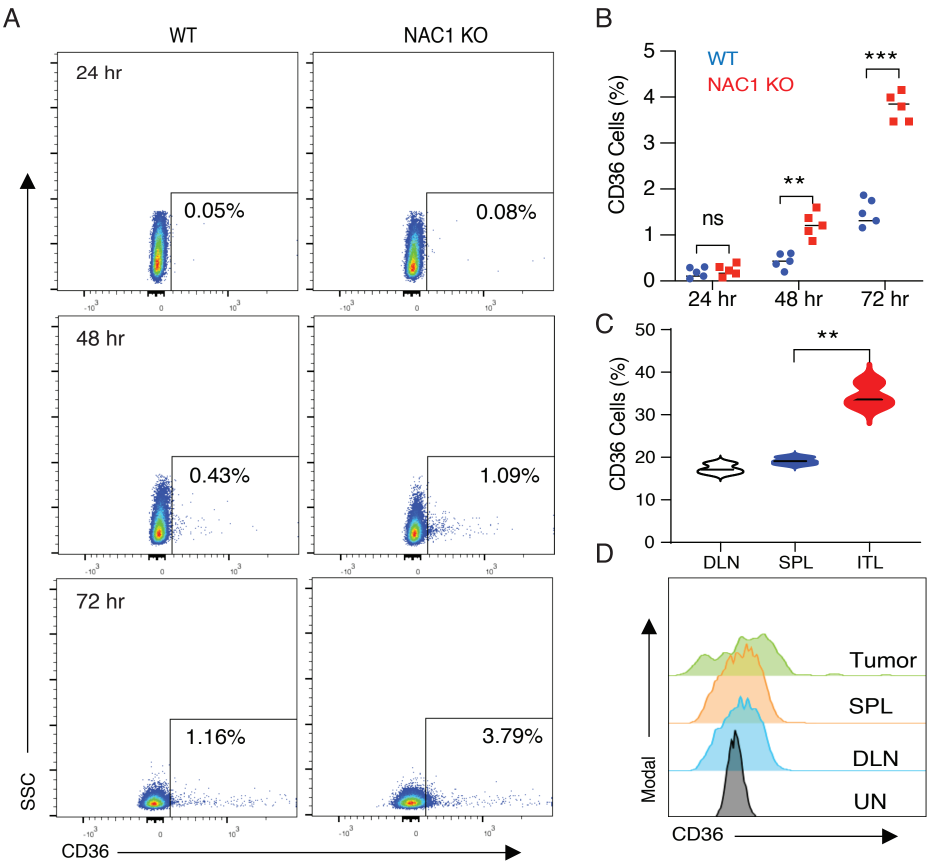

Supplemental Figure 3

A

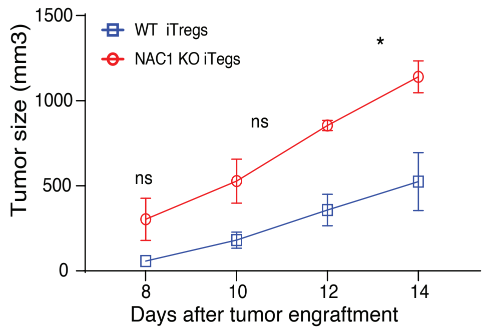

B

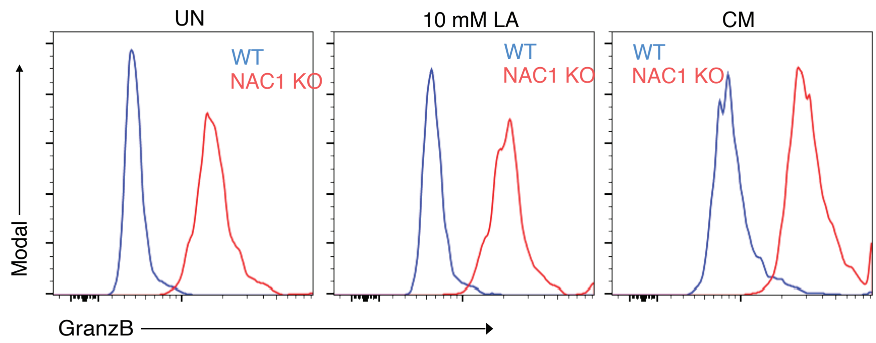

Supplemental Figure 4

A

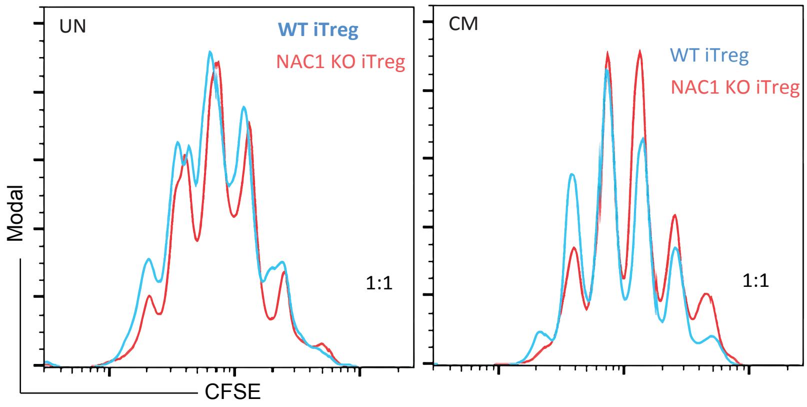

Supplemental Figure 5

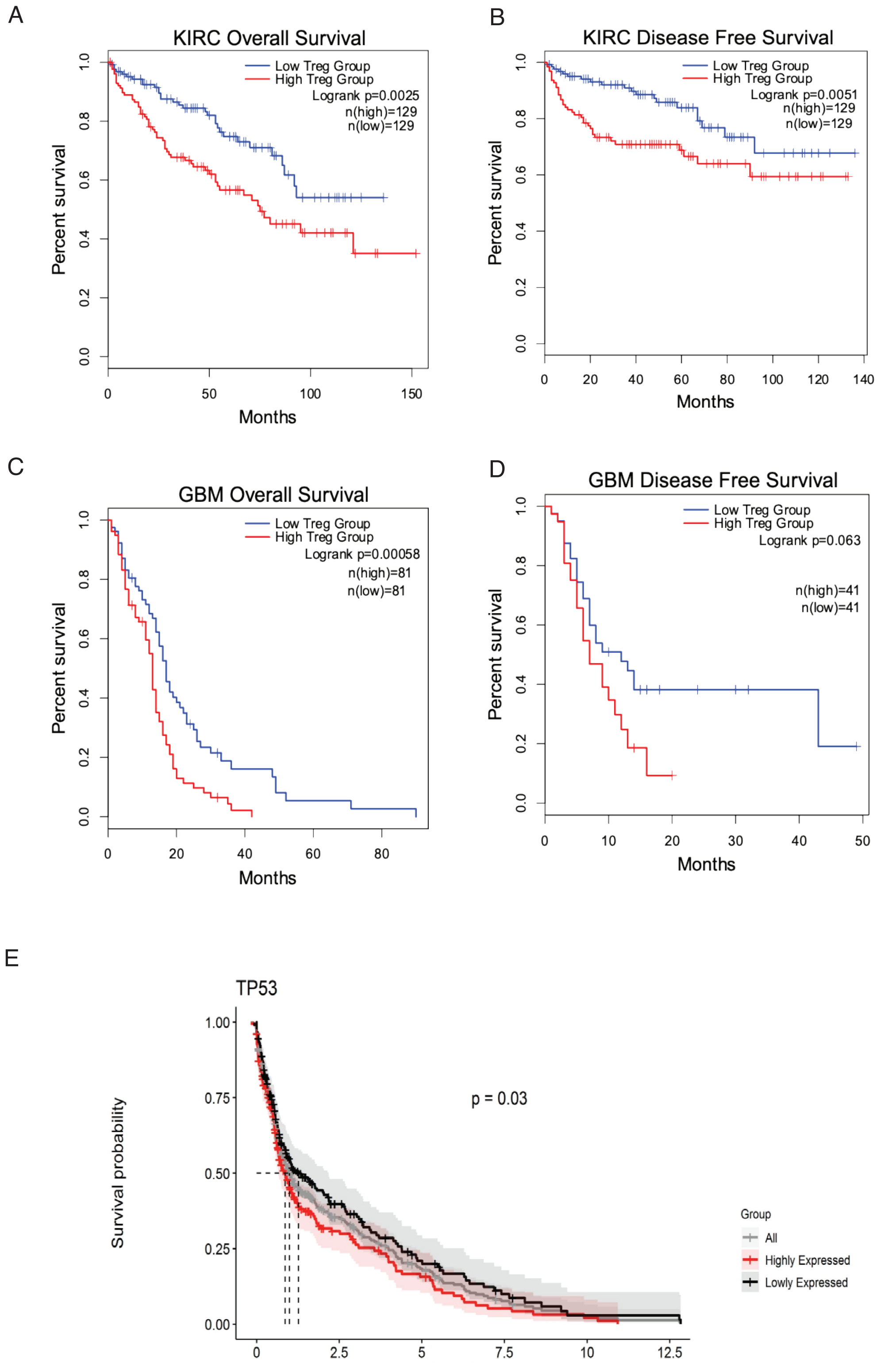

Supplemental Figure 6

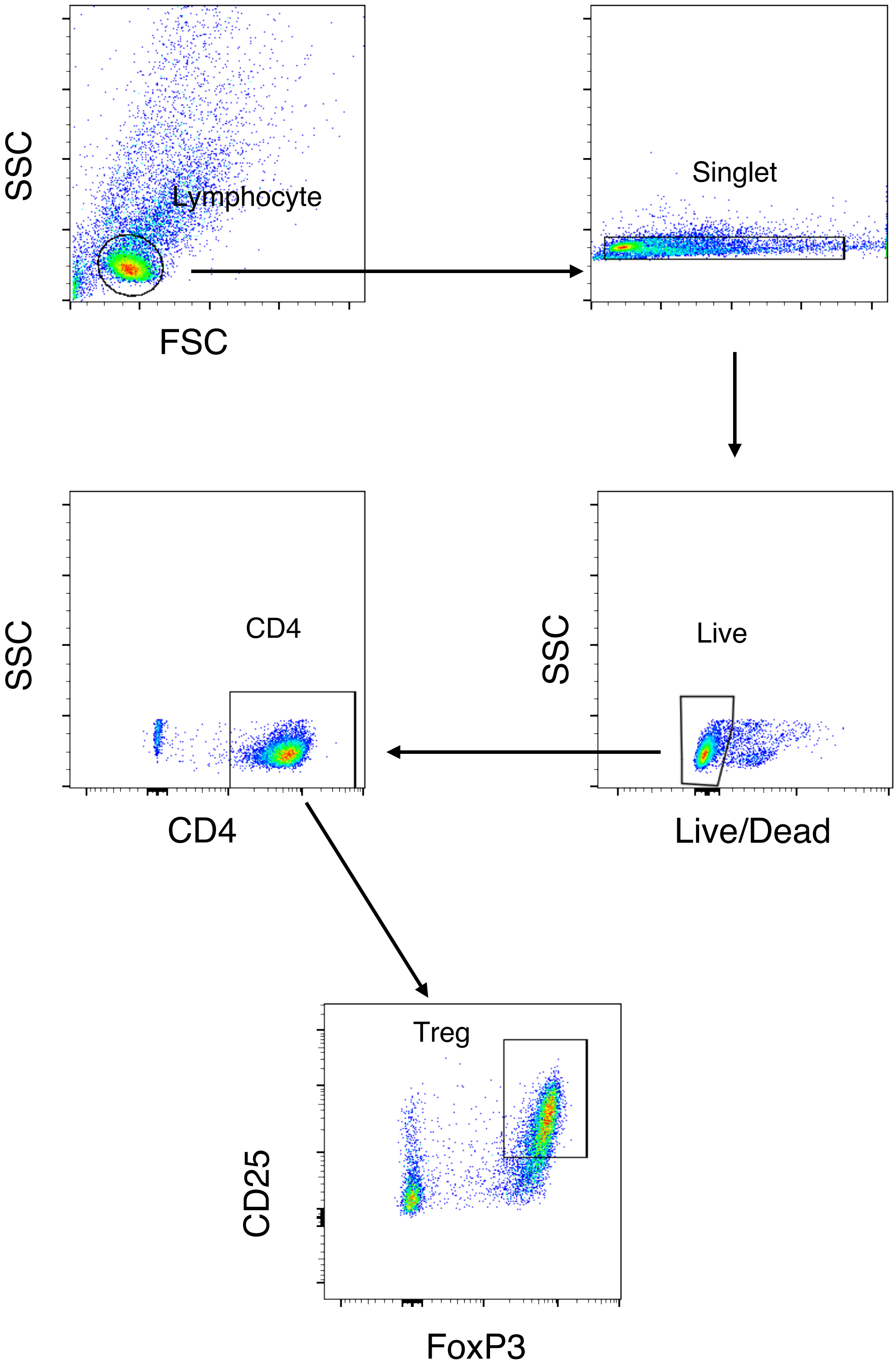

Supplemental Figure 7

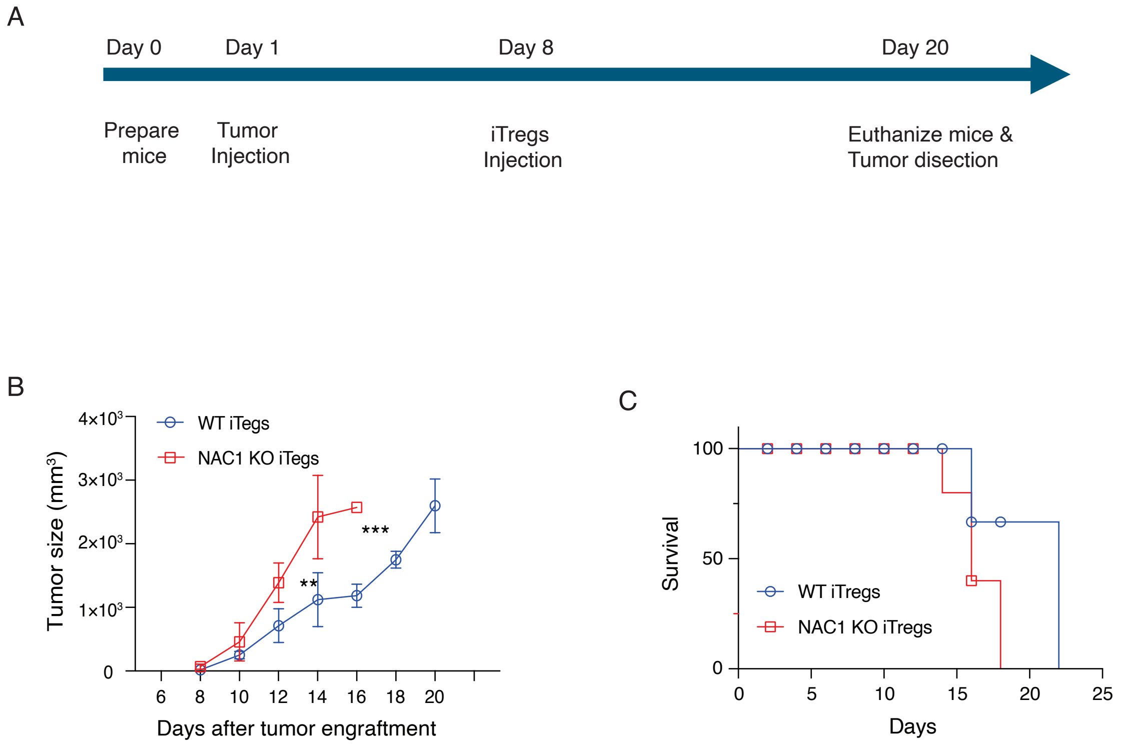

Supplement: Supplemental data [file jciinsight-10-186000-s020.pdf]
